# Integrating 2D Nanomaterials into Liquid Crystals for Multifunctional Nanocomposite Films

**DOI:** 10.1021/acsomega.6c06218

**Published:** 2026-08-17

**Authors:** Urice N. Tohgha, Kyung Min Lee, Michael A. Susner, Michael E. McConney, Peter R. Stevenson

**Affiliations:** † Air Force Research Laboratory, Materials and Manufacturing Directorate, 33319 Wright-Patterson Air Force Base, Dayton, Ohio 45433, United States; ‡ Azimuth Corporation, a Core4ce LLC, 2079 Presidential Dr. #200, Fairborn, Ohio 45342, United States

## Abstract

We report a facile strategy to exfoliate and disperse transition metal dichalcogenides (WS_2_, MoS_2_, VS_2_) and MXene (Ti_3_C_2_T_*x*_) directly within liquid crystals (LCs), yielding stable multifunctional nanocomposites. The multifunctional composite films exhibited unique properties, highlighted by the nanomaterial-mediated cholesteric-to-isotropic transition in the LC/Ti_3_C_2_T_*x*_ composite films at lower applied fields than the LC film alone. In addition, we demonstrated the preparation of free-standing LC/Ti_3_C_2_T_*x*_ composite films having both distinct LC and nanomaterial optical characteristics. Our solvent-free approach also enabled alignment-layer-free blade coating of birefringent composite films without postsynthesis processing. Significant polarization-dependent absorption was observed for LC/MoS_2_ films due to the anisotropy of the composite. The facile film processing and composite film properties are appealing for the fabrication of next-generation optoelectronics.

## Graphical abstract

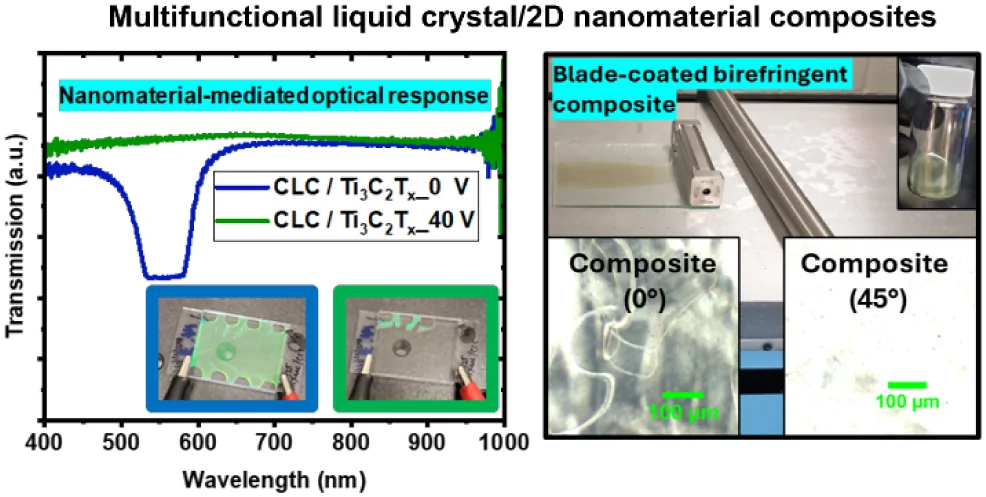

## Introduction

Liquid crystals (LCs) exhibit unique optical properties that may be further tailored by incorporating low-dimensional nanomaterials, thus yielding multifunctional nanocomposite thin films. For example, cholesteric liquid crystals (CLCs) can offer wavelength-selective optical reflection of circularly polarized light determined by the CLC pitch.^1–4^ There have been extensive reports on the photonic,^5,6^ thermal,^7,8^ and electro-optic properties of LCs,^9,10^ but limited efforts have been devoted to taking advantage of the intrinsic properties of two-dimensional (2D) nanomaterials to modulate or enhance such characteristics of LCs.^11^

2D materials, including transition metal dichalcogenides (TMDCs)^12–16^ and transition metal carbides and nitrides (MXenes),^17–19^ have been shown to exhibit appealing material characteristics including optical nonlinearity,^20^ magneto-optical properties,^21,22^ high electrical conductivity,^19^ and high thermal conductivity.^14^ Integrating these materials into functional liquid crystal composites offers exciting opportunities for developing novel materials^23–25^ and enhancing the performance of optical filters, polarizers, and nonlinear optics. LCs also have the potential to organize and/or orient incorporated nanomaterials, such as two-dimensional (2D) materials, resulting in tailorable optical behavior such as induced chirality.^26–29^

However, creating stable colloidal dispersions of near-pristine 2D nanomaterials within LC media has been challenging due to inherent compositional incompatibilities of the materials involved. Traditional methods for preparing such composites are often solvent-based, using, in some cases, hazardous high-boiling point solvents that pose challenges during composite preparation.^30^ Aqueous-based approaches are widely utilized in the preparation of lyotropic LCs such as in the case of graphene oxide.^31–33^ However, the removal of water is required for composite preparation. Gogotsi and coworkers have prepared lyotropic liquid crystals of 2D nanomaterials in various organic solvents.^18^ However, scalability of this approach is limited by the high concentration of nanomaterials and the aspect ratio requirements of the 2D nanomaterials, making it less environmentally friendly.^34^ Ligand functionalization has also been employed to disperse premade nanomaterials in LCs but it is an additional time-consuming step.^24^ We recently demonstrated the direct exfoliation of 2D nanomaterials in monomers facilitating film processing without solvents and ligand functionalization.^35,36^ Some of the recently reported procedures for exfoliating 2D nanomaterials into LCs are time-consuming and laborious.^37^ Overall, there is a growing need for simpler, more environmentally friendly, and, more importantly, scalable fabrication techniques that can yield high-quality multifunctional composite films.^38^

Here, we present a robust, in situ exfoliation of a diverse library of 2D materials—including TMDCs (semiconducting MoS_2_ and WS_2_ as well as metallic VS_2_) and an MXene (metallic Ti_3_C_2_T_*x*_)—directly within LCs in a one-step procedure (in some cases without additional solvents). We demonstrate that the LCs themselves can serve as effective exfoliation media, stabilizing the nanosheets without intermediate processing. This approach unlocks new functional modes: we report a low-voltage cholesteric-to-isotropic phase transition driven by internal Joule heating in MXene composites, and substantial polarization-dependent absorption in alignment-layer-free LC/MoS_2_ films. This methodology not only simplifies fabrication but also provides a versatile platform for scalable multifunctional optical coatings.

## Materials and Methods

Vanadium disulfide (VS_2_), Tungsten disulfide (WS_2_) and Molybdenum disulfide (MoS_2_) were prepared using the procedure described below.

Methyl methacrylate (MMA) monomer (Aldrich, 99%), %, Irgacure 651 (2,2-dimethoxy-1,2-diphenylethanone), were purchased from Aldrich. All chemicals were used as received.

### Procedure for Growing Bulk TMDC Materials^15^

The metal (Mo, V, W) components of the compounds were either sourced as metal foil, generally <100 μm in thickness or powders. In the latter case, the powders were first reduced at 200–300 °C under flowing H_2_ to remove any oxide contamination. The metal was combined with the chalcogen material (S) in the molar ratio M:X of 1:2.05. The excess chalcogen was present to ensure full reaction of the metal. I_2_ was added (quantity adjusted to ensure 1–2 atm pressure at reaction temperature) to act as a mineralizer/vapor transport agent to ensure reaction completion and ensure increased quality of the final crystalline material. These precursor materials were sealed, under vacuum, in a quartz ampule. The ampule reaction temperatures ranged from 700 to 1200 °C. Heating was applied at a rate of 30 °C per hour to the final reaction temperature, held at that temperature for periods ranging from 100 to 200 h, and cooled to room temperature at the same rate as heating. After cooling, the ampules were sectioned and the I_2_ was let to evaporate in a fume hood until it was completely dispersed. The final crystals were then removed from the quartz ampule and used for further processing.

### Procedure for Preparing Ti_3_C_2_T_*x*_ MXene

Synthesis of Ti_3_C_2_T_*x*_ was carried out according to the procedure in previously published work.^39^

### 14-Micron Cell Preparation According to Lee et al.^40^

Aligned cells were prepared using indium tin oxide-coated glass slides (Colorado Concepts, Loveland, CO, USA). The glass substrates were cleaned with acetone and methanol and then treated with air plasma for 30 s. The substrates were spin-coated with Elvamide. The alignment layer was rubbed with a cloth, and cells were constructed to ensure planar antiparallel alignment conditions (aligned cell). Cell gaps were controlled by mixing glass rod spacers (15 μm thickness) into an optical adhesive (NOA 65, Norland, Jamesburg, NJ, USA). The thickness of the cells was measured using an optical method based on the interference pattern of reflected light by the glass substrates in each empty cell.

### Microscopy

Transmission electron microscopy (TEM) images were collected using a Talos 200 kV microscope.

### Spectroscopy

Absorption spectrophotometry was performed using a Cary 5000 UV–vis-NIR spectrophotometer (Agilent). Transmission spectra were taken using an Ocean Optics spectrometer.

### Thermogravimetric Analysis (TGA) Characterization

Thermogravimetric analysis was carried out using a TA Instruments\TRIOS\TGA550.

### Polarized Optical Microscope (POM)

Polarized optical microscopy was carried out using a Nikon Eclipse 50i Pol.

### Blade Coating

Blade coating was performanced using an Auto Film Coater w/Heatable Vacuum Bed (12″W × 24″L) and 250 mm. Doctor Blade—MSK-AFA-II-VC-FH. 2D material/LC mixture was placed on a glass substrate and the applicator of appropriate thickness was used to make films of varying thicknesses (100–1000 μm). Coating was done at speeds of 15 mm/sec.

### Procedure for 2D Material Exfoliation in LC

The starting source powder was weighed and transferred to a 20 mL glass vial. Liquid crystal was used as the liquid medium as described in the Supporting Information. A bath sonicator (Digital Ultrasonic Cleaner with Variable Power, Elma) with water set to a temperature at the isotropic point of the LC (60 °C). Figures S1–S3 show details of sample preparation.

## Results and Discussion

Thin-film processing was carried out directly from the as-prepared colloidal dispersions following the exfoliation step (as shown in Figure 1). Briefly, bulk powders of 2D materials were added to a LC mixture, and exfoliation of 2D nanomaterial was achieved via mild sonication carried out directly in the LC mixture without the use of added solvents in some cases. For nematic LC/2D nanomaterial composites, film processing (i.e., UV-curing of blade coated films) was performed directly from the exfoliated material (details provided in the Supporting Information). For the CLC nanocomposites, chiral dopants were added prior to film processing to achieve the desired CLC reflection characteristics. Here, CLCs possess a helical, layered structure formed by chiral molecule additives that can rotate in orientation along a pitch axis. By varying the dopant concentrations, films with different reflection wavelengths were obtained, while maintaining the same content of 2D nanomaterials. Figures S1–S3 show detailed preparation protocols for the CLC/2D nanomaterial composites. Absorption spectra of the 2D nanomaterials in solution are shown in Figure S4. The content of 2D nanomaterial in the composite was determined by thermogravimetric analyses (TGA) and the curves are shown in Figures S5–S7, corresponding to 3.7%, 3.5%, and 3.8% for Ti_3_C_2_T_*x*_, WS_2_, and VS_2_ composites, respectively. TGA samples were 7 mg of polymerized drop cast composites. Polarized optical microscope (POM) images of the CLC composites are shown in Figure S8. The chemical structures of the LCs (RM82 and 5CB) and chiral dopants (S1011 and S811) are shown in Figure 1a, the LC/2D nanomaterial colloidal dispersions are shown in Figure 1b (black isotropic and green liquid crystalline states), and an example of a resulting composite thin film is shown in Figure 1c. Transmission electron microscope (TEM) images of the nanomaterials used in this work are shown in Figure 1d. Ti_3_C_2_T_*x*_ and VS_2_ nanoflakes ranged in lateral size from 50 to 250 nm. WS_2_ and MoS_2_ nanoflakes ranged in lateral size from 75 to 200 nm.

**Figure 1:**
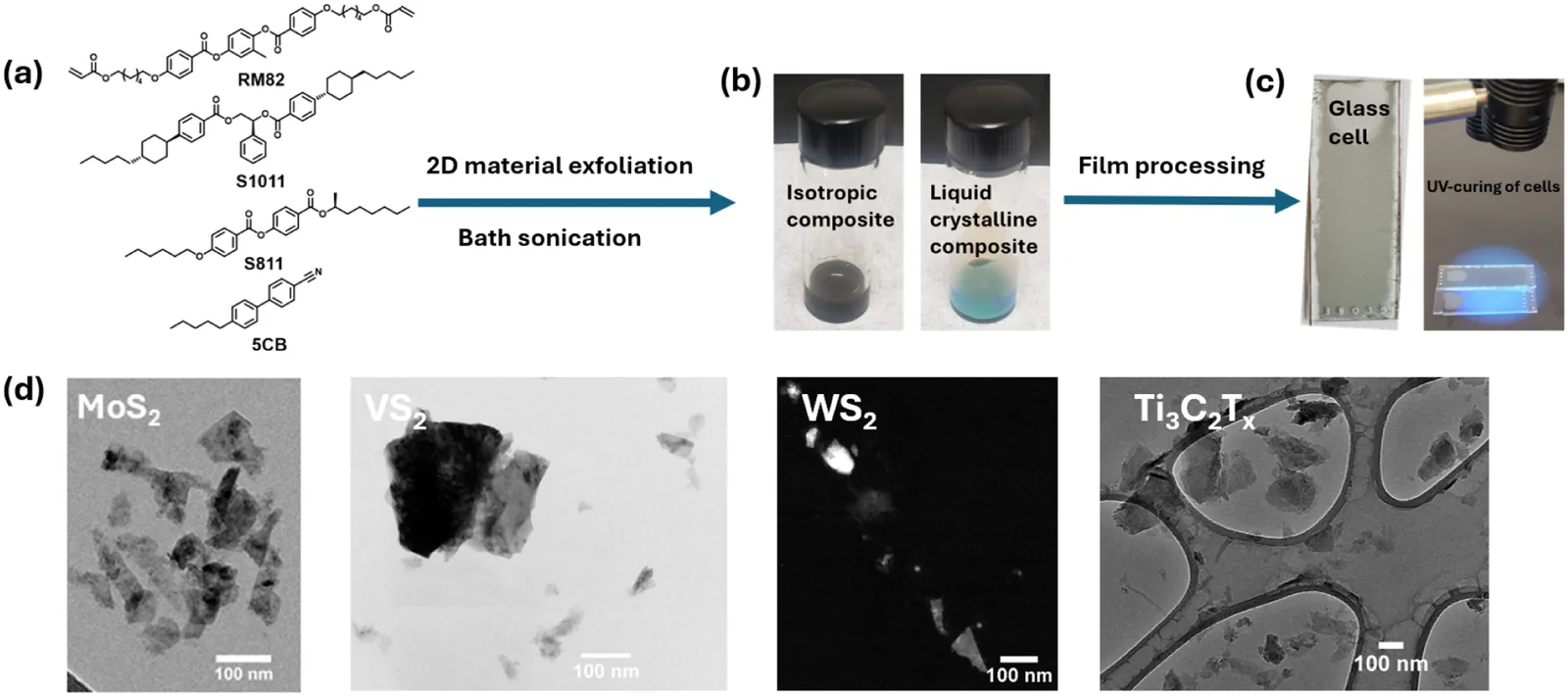
(a–c) Scheme of nanocomposite preparation involving LC/2D material colloidal dispersion and thin film processing. The vials in (b) are a representative sample at the isotropic (black, due to the 2D material) and liquid crystalline state (green). (c) Composite in a glass cell and UV curing of cells. (d) TEM images of the exfoliated 2D nanomaterials used in this work.

### 2D Nanomaterial-Mediated Electro-Optic Response

The incorporation of Ti_3_C_2_T_*x*_, WS_2_, and VS_2_ into CLCs influenced the electro-optic (EO) response of the LC films, as shown in Figure 2. A pure CLC mixture in a 14 μm thick planar cell was first characterized at different voltages (i.e., 0 and 40 V) as a reference (see Figure 2a). The film became opaque at 40 V due to the expected reorientation of LCs into a focal-conic state characterized by reduced optical transmission.^41^ Similar EO characterization was performed on CLC composite films containing the three different 2D nanomaterials as shown in Figure 2b, c, and d for Ti_3_C_2_T_*x*_, WS_2_, and VS_2_, respectively. At 40 V, all samples except CLC/Ti_3_C_2_T_*x*_ show the focal-conic, low optical transmission state. Interestingly, the CLC/Ti_3_C_2_T_*x*_ film switched to an optically transparent state with the application of 40 V. This was attributed to the intrinsic electrical conductivity of Ti_3_C_2_T_*x*_, which mediated the EO properties of the LC film through joule heating. The cell temperature was observed to rise above the transition temperature of the LC mixture (>50 °C), thus inducing the optical transparent state. Figure S9 shows temperature measurements of a CLC cell and CLC/Ti_3_C_2_T_*x*_ cell at the optically reflective and transparent states. While the CLC film temperature remains the same (23 °C), the temperature of the composite film changes dramatically from 24 to 60 °C at the optically reflective and transparent states, respectively. Upon removal of the electric field, the transparent CLC/Ti_3_C_2_T_*x*_ sample returned to its optically reflective state more slowly (>5 s) compared to the CLC film (<5 s). While the use of MXenes as Joule heaters in fabrics^42^ and films^18,43^ has been explored, there are limited reports on using these materials in composite films for modulating the optical behavior of LCs. As a representative example, Feng and coworkers deposited a blue phase LC film on a separate lyotropic MXene film using multiple steps, including surface functionalization of both the substrate and MXene.^44^ Their work also required specific nanoflake sizes and high concentration of MXene to achieve the LC phase. In contrast, our work used a single composite film prepared via a one-step process without surface functionalization. More importantly, liquid crystalline MXene was not required in our case, and no postexfoliation processing was performed (e.g., size-selective precipitation).

**Figure 2:**
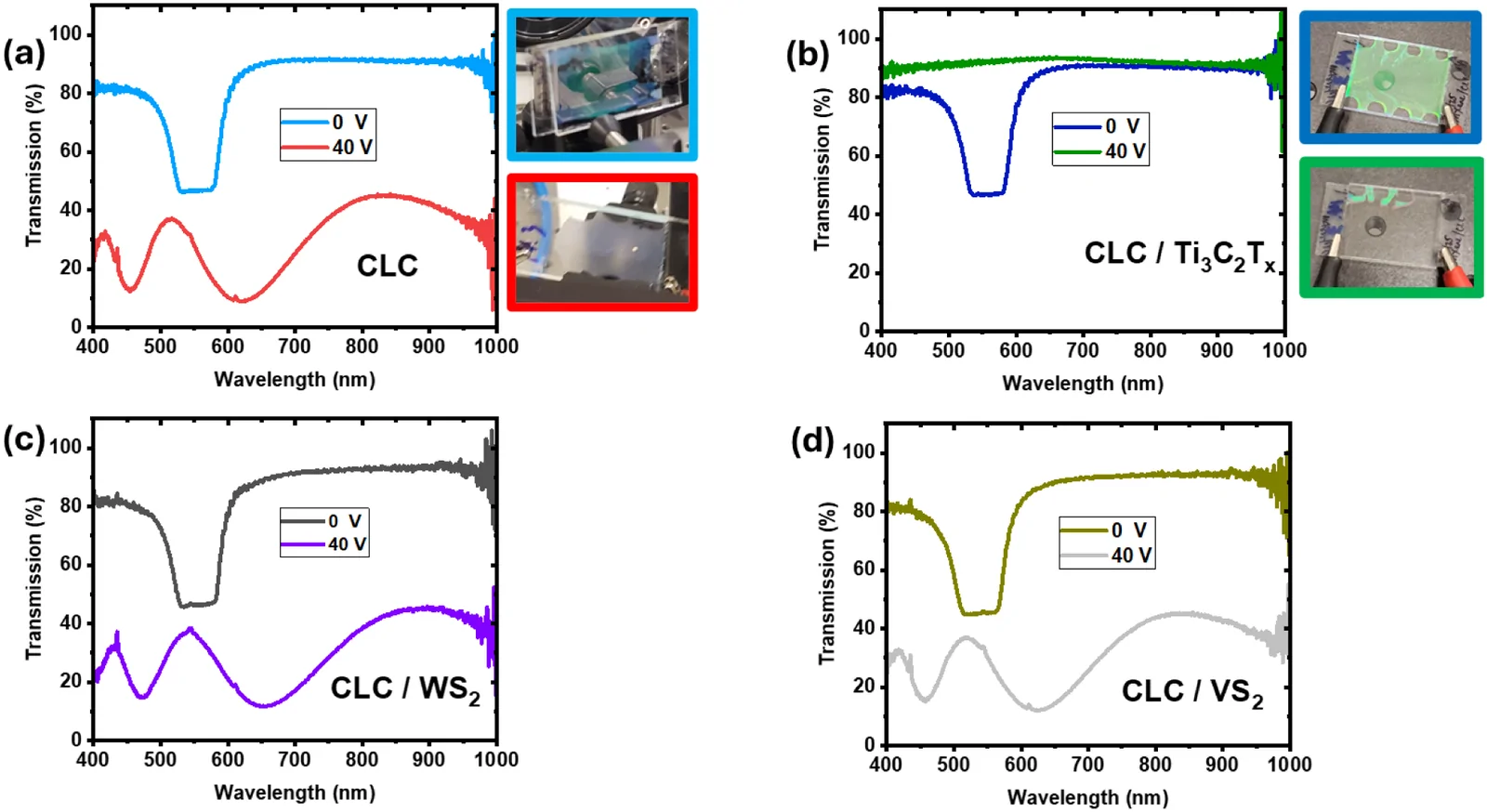
(a) Electro-optic properties of a pure CLC film with no 2D nanomaterials, (b) CLC/Ti_3_C_2_T*_x_* composite film, (c) CLC/WS_2_ composite film, and (d) CLC/VS_2_ composite film at 0 and 40 V at 1 kHz. Representative images of films are shown at 0 and 40 V and the color of the boxes around the films correspond to the color of the spectra. Films are not shown for (c) and (d) because these are similar to those shown in (a).

The EO responses of WS_2_ and VS_2_ composite films were found to be similar to those of the pure CLC film at the same voltage range (i.e., 0–40 V). However, the VS_2_ film exhibited electrical breakdown when the voltage increased to 60 V (Figure S10). The optical response of the LC film stems from the effect of applied field. The materials have different intrinsic properties (VS_2_ has magnetic and metallic properties while WS_2_ has semiconductor properties) and so it is reasonable to assume that the effect of electric field will be different for both materials. However, further work will be needed to better understand why this breakdown was observed at higher voltage. The pure CLC film and CLC/WS_2_ film still showed identical EO properties at higher voltages (i.e., 60–80 V) and both films were transparent at 80 V (see Figure S10) due to the samples transitioning to the homeotropic state. The absence of a nanomaterial-induced effect for LC/WS_2_ composites suggests that these could be particularly suitable for applications that exploit the optically reflective characteristics of CLCs in combination with the intrinsic optical reducing behavior of WS_2_, making them promising candidates for light attenuation applications.^20^

### Ti_3_C_2_T_*x*_ Nanocomposite Polymer Films with Fixed Optical Behavior

Based on the EO responses, we envisaged that the conductive properties of LC/Ti_3_C_2_T_*x*_ composites can be utilized in polymer films for optical coatings having dual characteristics (e.g., electrical conductivity and optical reflection), thus representing quintessential multifunctional films. This concept was realized through the use of LC polymers, wherein Ti_3_C_2_T_*x*_ was incorporated via a similar one-pot exfoliation approach (described above), employing only the LC monomer (RM82) and chiral dopants (S1011, S811) (see Figure S11). Here, the 2D nanomaterial was directly exfoliated in the LC polymer system with a comonomer in solution (methyl methacrylate, MMA), which is compatible with the LC acrylate monomer. Following exfoliation, the MMA monomer evaporated and did not affect subsequent film processing.^36^ While LC polymers have been explored,^45,46^ there are no literature reports (to our knowledge) on the direct incorporation of conductive MXenes into such polymers to achieve multifunctional films. Films exhibiting LC optical characteristics in the visible and infrared regions were successfully fabricated with incorporated Ti_3_C_2_T_*x*_, as shown in Figure 3. The spectra of pure CLC and CLC/Ti_3_C_2_T_*x*_ films are shown in Figure 3a and b for the visible wavelength regime. The characteristic Ti_3_C_2_T_*x*_ absorption feature at ∼790 nm was observed (see Figure S4). The CLC peak around 532 nm was significantly broadened for the composite relative to the pure CLC film (see Figure 3a), indicating a nanomaterial-induced modification of LC optical response. Furthermore, large-area (7-inch wide and 80 μm thick) film of CLC Polymer/Ti_3_C_2_T_*x*_ was successfully made (see Figure 3b), demonstrating the scalability of this approach. A composite film with a CLC notch in the infrared (IR) region (∼1500 nm) was also prepared as shown in Figure 3c, and the characteristic Ti_3_C_2_T_*x*_ absorption peak can be observed as well. The spectra in Figure 3c are shown at a different wavelength scale from the 3a and b, to highlight both the Ti_3_C_2_T_*x*_ absorption feature and the CLC polymer peak in the IR. This conceptual approach could be extended to films with optical characteristics in the midwave infrared, and such multifunctional films are likely to be of interest for optoelectronic applications.

**Figure 3:**
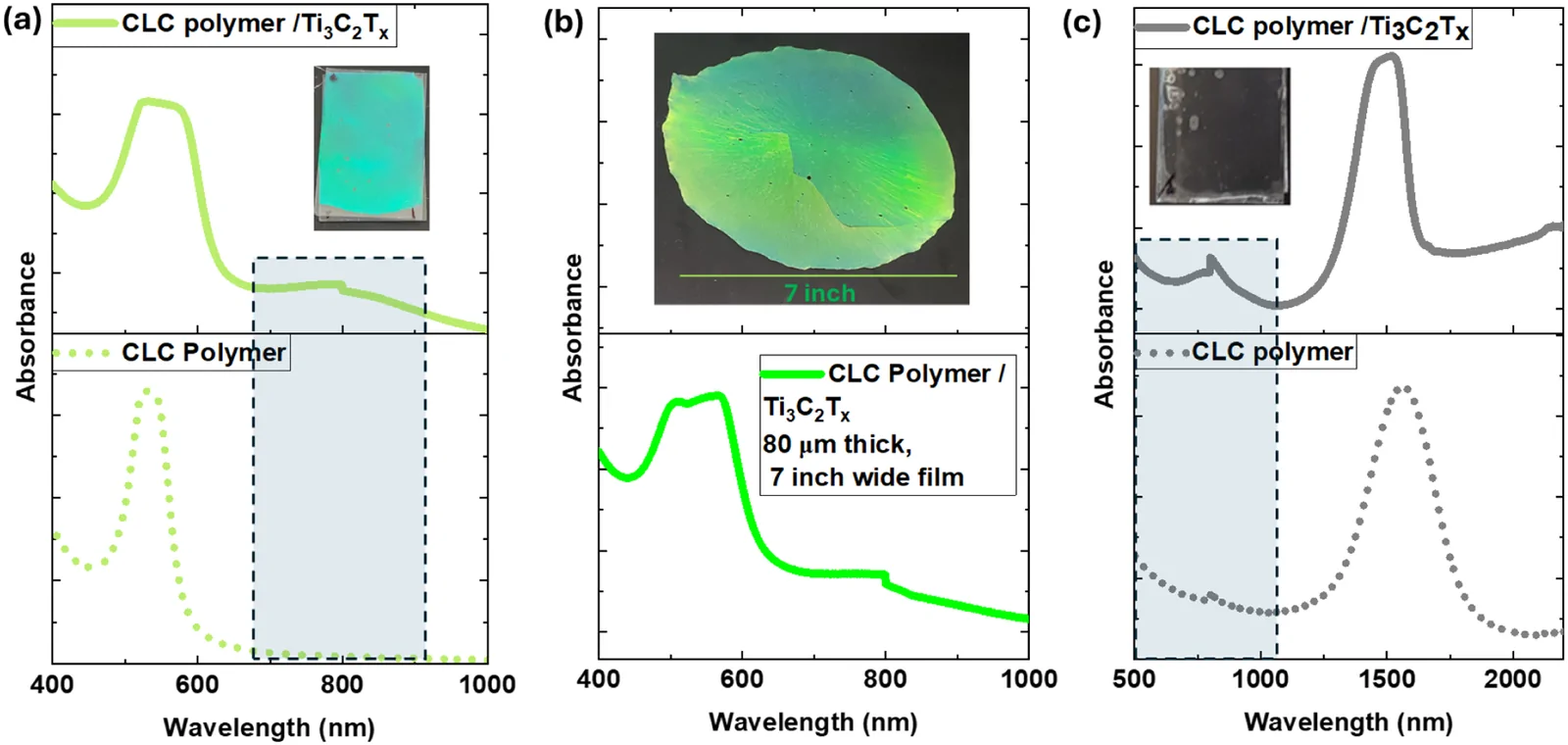
(a, b) CLC polymer/Ti_3_C_2_T_x_ and CLC polymer exhibiting a CLC peak in the visible region. (b) 7-in.-Wide free-standing composite CLC polymer/Ti_3_C_2_T_x_ film and corresponding spectrum. (c) CLC polymer/Ti_3_C_2_T_x_ and CLC polymer showing the CLC peak in the infrared. The Ti_3_C_2_T_x_ absorption feature at ∼790 nm is highlighted by the dashed rectangle in (a) and (c). An artifact can be seen at 800 nm from the spectrometer.

### Alignment Layer-Free, Blade-Coated Birefringent Composite Films

Our solvent-free approach allowed for the blade-coating of nematic LC-based composites right after 2D nanomaterial exfoliation. Samples were prepared as described above without the addition of chiral dopants (compare Figure S3). Nanomaterials exhibiting well-defined absorption peaks were selected to probe potential LC-induced optical characteristics of the resulting composite films. Figure 4a illustrates alignment layer-free birefringent nematic LC/WS_2_ composite films. Here, nematic LCs represent molecules with long-range orientational order but no positional order (as compared to CLCs). Blade coating was performed at a speed of 15 mm/s (see the Supporting Information for more details). Optical transmission spectra of blade-coated films with varying thicknesses (100 μm, 250 μm, and 1000 μm) are presented in Figure 4b, showing the expected film thickness-dependence on optical transmission. The WS_2_ exciton peak at ∼627 nm can be observed for all films.^36^ POM images of the 250 μm film at different angles (0°, 45°, and 90°) showed distinct birefringent features (see Figure 4c). The versatility of the process was further demonstrated by blade-coating a 250 μm thickness nematic LC/MoS_2_ composite film. POM images of the composites were also taken at different angles as shown in Figure 4d. A spectrum of the corresponding film showed characteristic MoS_2_ exciton peaks at 607 and 669 nm (Figure 4e).^36^ Such composites have appealing properties such as birefringence, as well as the intrinsic optical characteristics of the incorporated 2D nanomaterials. Moreover, the simplicity of processing demonstrated here helps to facilitate the fabrication and broadened applicability of such composite systems for multifunctional thin films.

**Figure 4:**
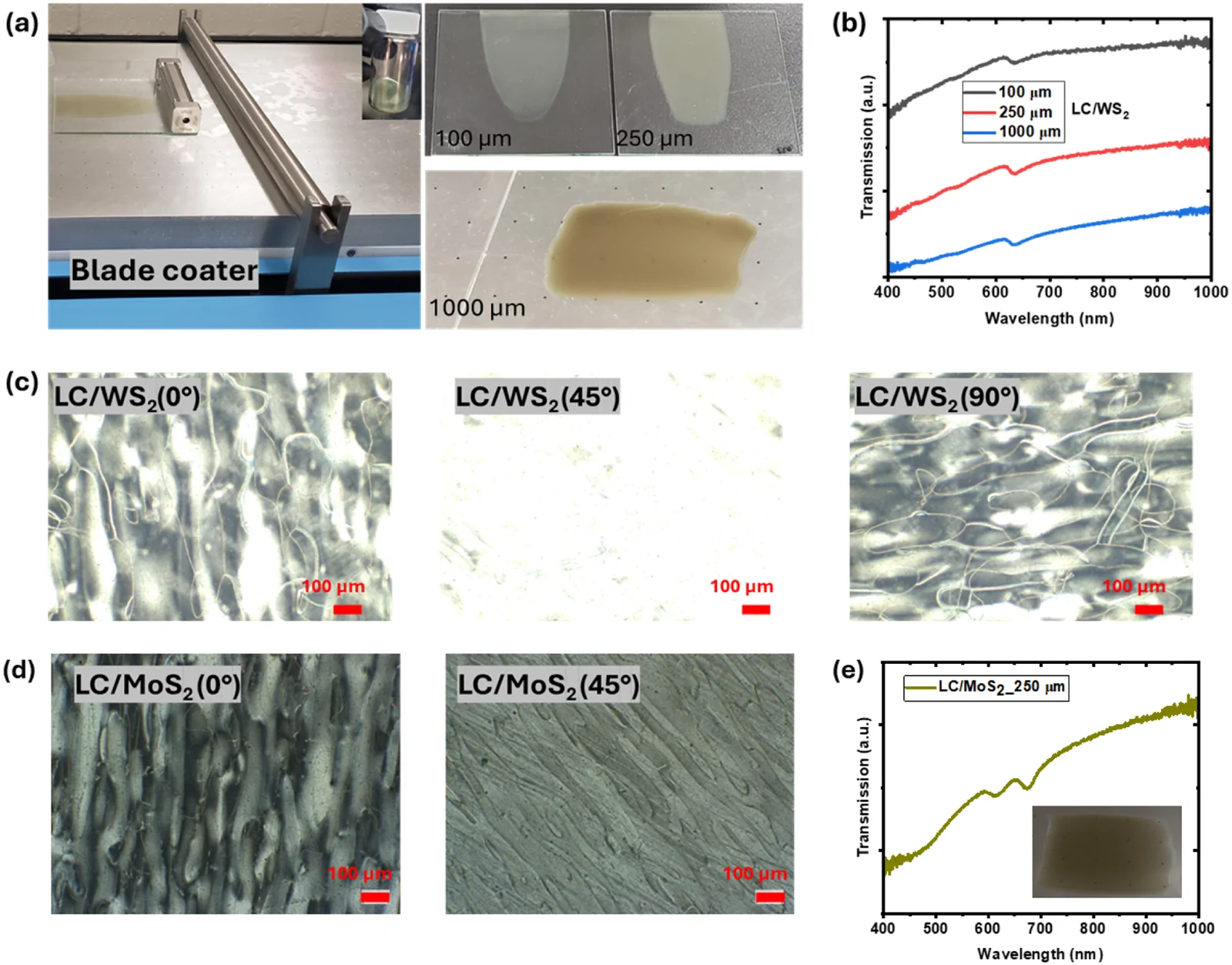
(a) Alignment layer-free, blade-coated birefringent LC/WS_2_ composite films with various thicknesses (100 μm, 250 μm, and 1000 μm). (b) Transmission spectra of the different LC/WS_2_ films. (c) POM images of the 250 μm film at 0°, 45° and 90° angle of incidence. (d) POM images of the 250 μm LC/MoS_2_ composite film at 0° and 45° angle of incidence and (e) the corresponding transmission spectrum. (f) Inset image of the film. POM images taken at 10× magnification. Scale bars are 100 μm in length.

It is important to note that the geometric anisotropy of the given nanomaterial may contribute to the birefringence of the composite. To examine this effect, polarized light measurements were conducted with the aim of probing potential modification to the intrinsic optical behavior of the nanomaterials. Linearly polarized light was used to characterize aligned films by carrying out angle-dependent measurements.^47^ Specifically, LC, LC/MoS_2_, and LC/WS_2_ films were characterized by analyzing angle-dependent *p*-polarization (i.e., the electric field is parallel to the plane of incidence) and *s*-polarization (i.e., the electric field is perpendicular to the plane of incidence), as shown in Figure 5. The transmission spectra for both *p*- and *s*-polarized light were obtained at various angles (0°, 45°, and 60°) for pure LC (Figure 5a–c), LC/MoS_2_ (Figure 5d–f), and LC/WS_2_ (Figure 5g–i) films.

**Figure 5:**
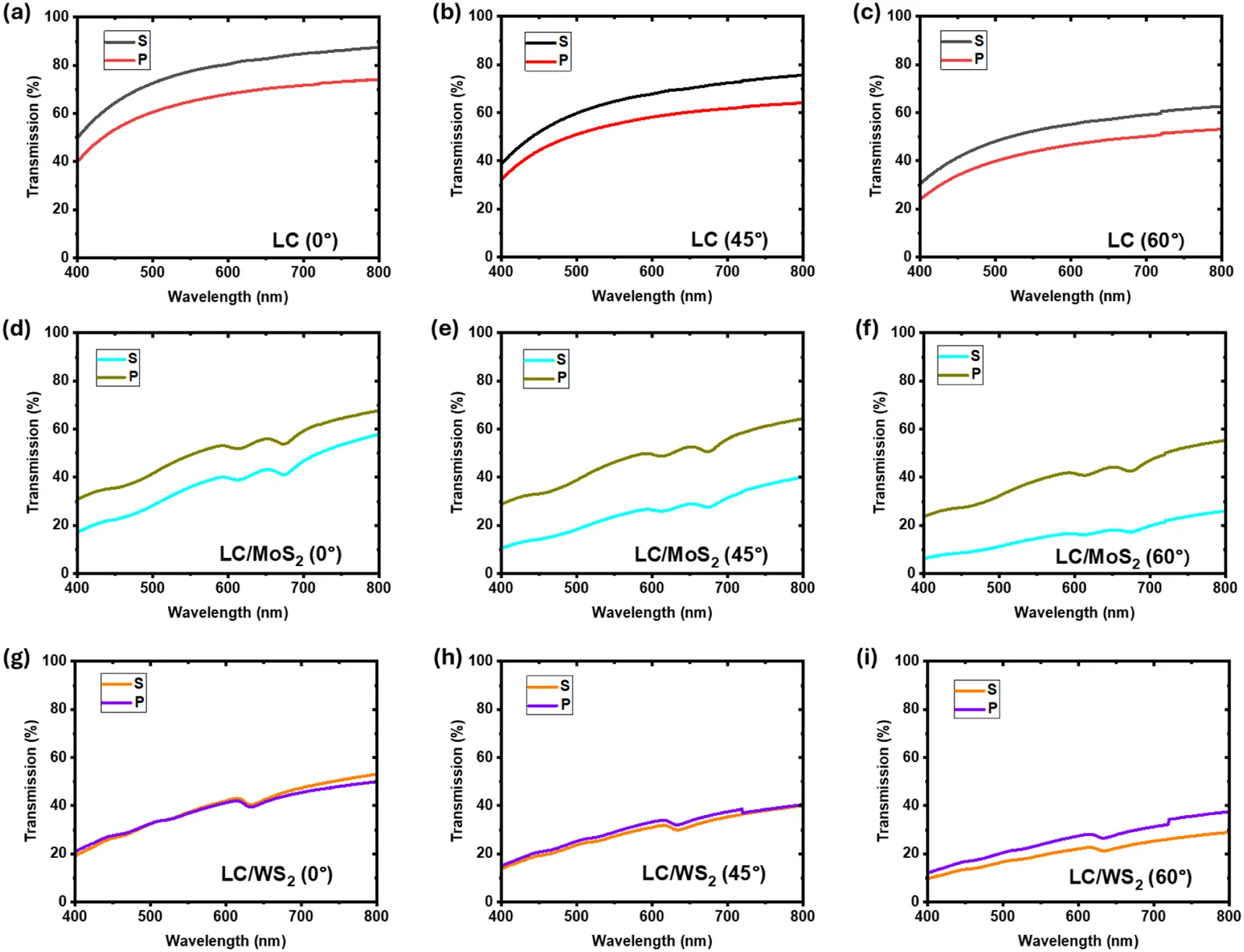
(a–c) *P*- and *s*-polarized transmission (T) spectra for a pure LC film, (d–f) a LC/MoS_2_ film, and (g–i) a LC/WS_2_ film. Each film was measured at 0°, 45° and 60° angles of incidence.

The difference in *p*- and *s*-polarized signal intensities (ΔT%) were comparable at 0° for LC (ΔT% = 11.7%, Figure 5a) and LC/MoS_2_ films (ΔT = 12.9%, Figure 5d). However, distinct differences are observable at higher angles. At 45°, ΔT% for LC/MoS_2_ was 22.9%, more than double that of the pure LC film (ΔT% = 9.9%). A similar trend was observed at 60° angle of incidence, where ΔT% values of 8.7% (Figure 5c) and 25.2% (Figure 5f) were obtained for LC and LC/MoS_2_, respectively. These results suggest a nanomaterial-induced effect, where the shape anisotropy and/or orientation of the nanoflakes influence the relative attenuation of *p*- and *s*-polarized light. However, the LC/WS_2_ composite showed very different results compared to LC/MoS_2_ films. At 0° angle of incidence, the difference between *p*- and *s*-polarized signal intensities was negligible (ΔT% = 0.6%, Figure 5g). Even at 60° angle of incidence, only a modest difference was observed (ΔT% = 5.6%, Figure 5i). This may arise from differences in the interaction and compositional compatibility of the LC matrix with WS_2_ versus MoS_2_, attributable to heterogeneous surface characteristics of such 2D nanomaterials (including crystallographic phase differences and chemical defects).^48^ Notably, *s*-polarized light was more attenuated than *p*-polarized light (more apparent at 60° angle of incidence) for both LC/MoS_2_ (Figure 5f) and LC/WS_2_ (Figure 5i) composites in contrast to the pure LC film (Figure 5c). There is thus a likelihood of an angle- and polarization-dependent optical nanomaterial effect that warrants further study. It is also important to point out the significant changes to the MoS_2_ exciton peaks at 607 and 669 nm under angle-dependent measurements. At 60°, the peaks became less defined for *s*-polarized light (Figure 5f) compared to the sharper peak profiles at 0° (Figure 5d). There is ongoing work to further explore the possibility of induced birefringence on the nanomaterial by the LC matrix.

## Conclusions

In summary, we have established a robust approach for the in situ exfoliation of 2D nanomaterials directly within liquid crystals without added solvents in some cases. This approach yields multifunctional nanocomposites with distinct, material-specific behaviors enabled by the intimate coupling between the LCs and the exfoliated nanosheets. Specifically, the CLC/Ti_3_C_2_T_*x*_ composites demonstrate a reversible cholesteric-to-isotropic phase transition driven by a percolation-enabled internal Joule heating. This volumetric heating mode offers a pathway to reconfigurable nanocomposite films that eliminate the need for external heating layers. We further demonstrated the preparation of free-standing, large-area CLC/Ti_3_C_2_T_*x*_ polymer films (i.e., 7-inches wide) illustrating the potential of larger scale optical coating applications. Nematic LC/2D nanomaterial composites were also prepared via alignment-layer-free blade coating. These films showed significant polarization-dependent absorption due to the geometric anisotropy of the composite. By successfully integrating the material characteristics of select 2D materials with the optical anisotropic behavior of representative liquid crystals, this work presents a simple, scalable pathway toward the fabrication of next-generation tailorable smart optical coatings and flexible optoelectronics.

## Supplementary Material



## Data Availability

The data that support this work are available from the corresponding author upon reasonable request.

## Notes

Preparation of CLC/Ti_3_C_2_T_*x*_; preparation of CLC/VS_2_; preparation of CLC/WS_2_; absorption spectra of 2D materials; TGA curve of LC/Ti_3_C_2_T_*x*_; TGA curve of LC/WS_2_; TGA curve of LC/VS_2_; POM images of composites; transmission spectra of CLC, CLC/WS_2_, and CLC/VS_2_ at 60 and 80 V; preparation of CLC polymer/Ti_3_C_2_T_*x*_ film (PDF)
