# Supplementary material for: Integrating 2D Nanomaterials into Liquid Crystals for Multifunctional Nanocomposite Films
Source: ACS Omega. 2026 Aug 17;11(34):52105–13. doi: 10.1021/acsomega.6c06218 (PMC13625096; doi:10.1021/acsomega.6c06218)
Supplement: Supplementary file 1 [file ao6c06218_si_001.pdf]

Supporting Information

Integrating 2D Nanomaterials into Liquid Crystals for Multifunctional Nanocomposite Films

Urice N. Tohgha[1,2], Kyung Min Lee[1, 2], Michael A. Susner[1], Michael E. McConney[1], and Peter R. Stevenson[1,*]*

[1]*Air Force Research Laboratory, Materials and Manufacturing Directorate, Wright-Patterson Air Force Base, Dayton, Ohio 45433, USA*

[2]*Azimuth Corporation, a Core4ce LLC, 2079 Presidential Dr. #200, Fairborn, Ohio 45342, USA*

*Email: urice.tohgha.ctr@us.af.mil

*Email: peter.stevenson.2@us.af.mil

Table of Contents

Liquid crystal structures and chiral dopants 3

Figure S1. Preparation of cholesteric LC/$Ti_3C_2T_x$ 3

Figure S2. Preparation of cholesteric LC/VS_2 4

Figure S3. Preparation of cholesteric LC/WS_2 5

Figure S4. Absorption spectra of 2D nanomaterials 6

Figure S5. TGA curve of LC/$Ti_3C_2T_x$ composite 7

Figure S6. TGA curve of LC/WS_2 composite 8

Figure S7. TGA curve of LC/VS_2 composite 9

Figure S8. POM images of composites 10

Figure S9. (a, b) Temperature measurements of the CLC cell at the reflective and transparent states respectively. (c, d) Temperature measurement of the CLC/$Ti_3C_2T_x$ cell at the reflective and transparent states respectively. 11

Figure S10. (a) Transmission spectra of CLC, CLC/WS_2 and CLC/VS_2 at 60V. (b) Depiction of CLC/VS_2 cell breakdown. (c) Transmission spectra of CLC and CLC/WS_2 at 80V. 12

Figure S11. Preparation of CLC Polymer/$Ti_3C_2T_x$ composite 13

Liquid crystal structures and chiral dopants

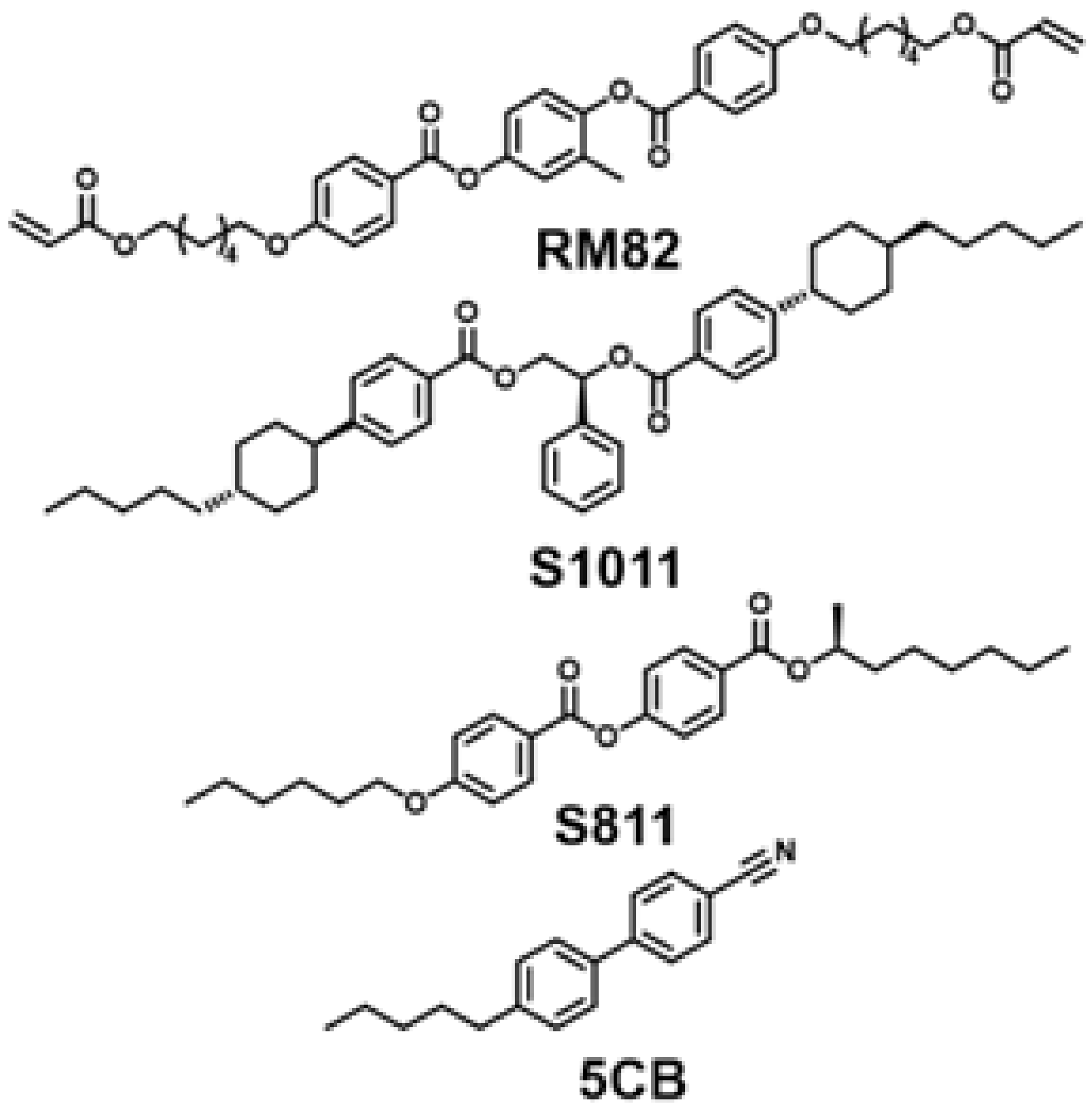

- 22 mg $Ti_3C_2T_x$
- 400 mg RM82
- 1600 mg 5CB
- Sonication 3 h, 60 C,

250 mg mixture
- Add chiral dopant S10 11 (10 mg), S811 (41.4 mg)
- Sonicate 3 mins, 60 C

- **Green mixture in LC state**

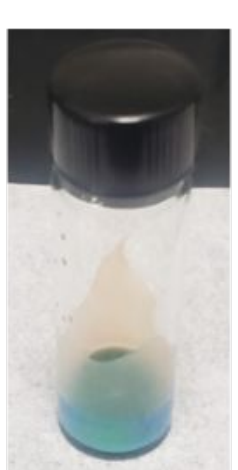

LC/$Ti_3C_2T_x$
(cholesteric)

Figure S1. Preparation of cholesteric LC/$Ti_3C_2T_x$

- 22 mg VS_2
- 400 mg RM82
- 1600 mg 5 CB
- Sonicate 3h, 60C,

250 mg mixture

- Add chiral dopant S10 11 (10 mg), S811 (41.4 mg)
- Sonicate 3 mins, 60 C

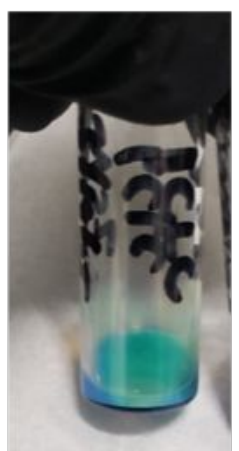

LC/VS_2
(cholesteric)

Figure S2. Preparation of cholesteric LC/VS_2

- 22 mg WS_2
- 400 mg RM82
- 1600 mg 5 CB
- Sonicate 3h , 60C,

250 mg mixture

- Add chiral dopant S10 11 (10 mg), S811 (41.4 mg)
- Sonicate 3mins, 60 C

- **Green mixture in LC state**

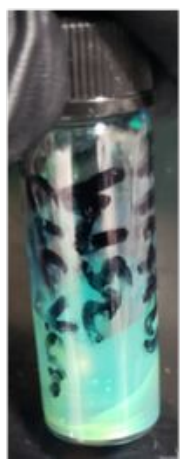

LC/WS_2
(cholesteric)

Figure S3. Preparation of cholesteric LC/WS_2

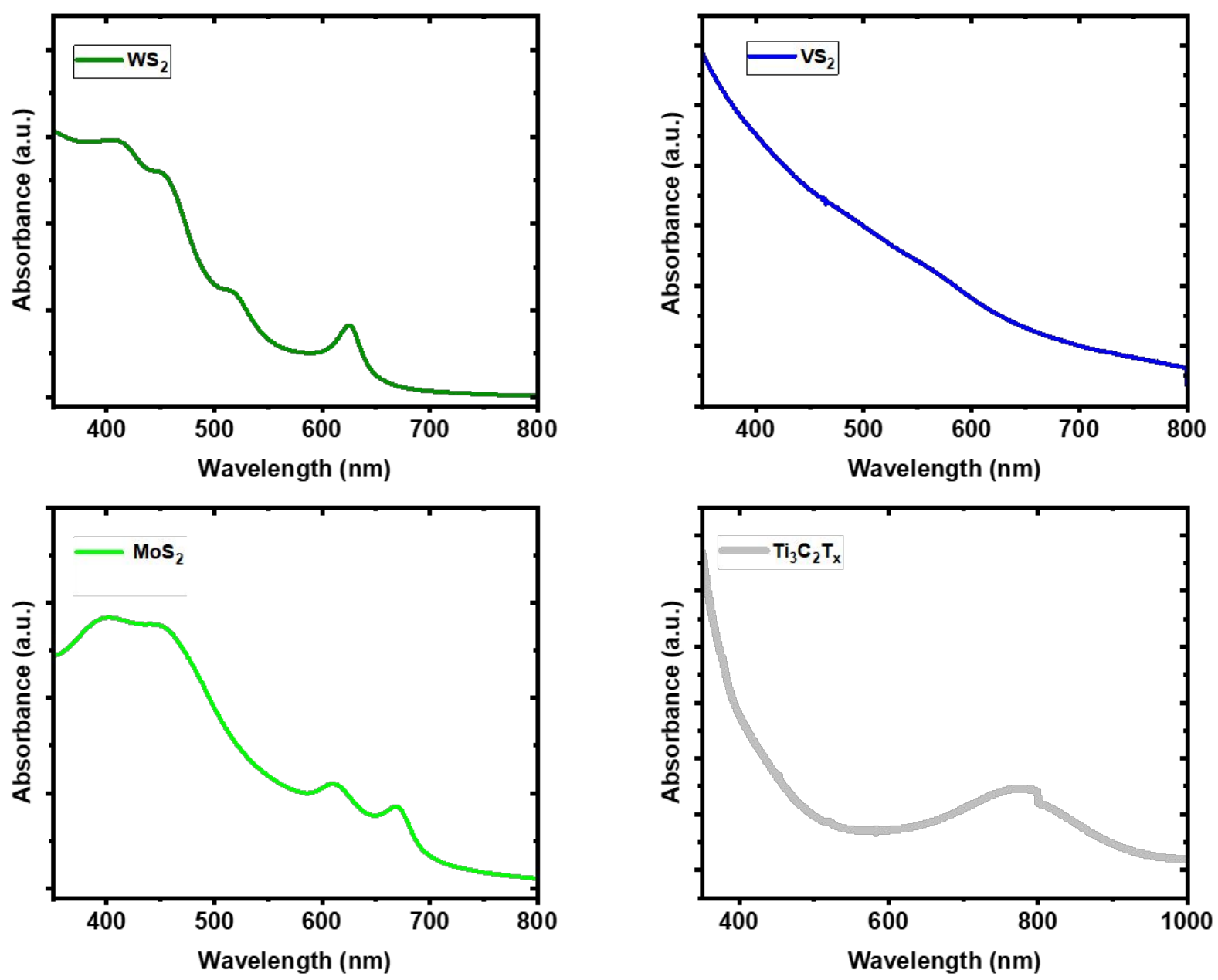

Figure S4. Absorption spectra of 2D nanomaterials

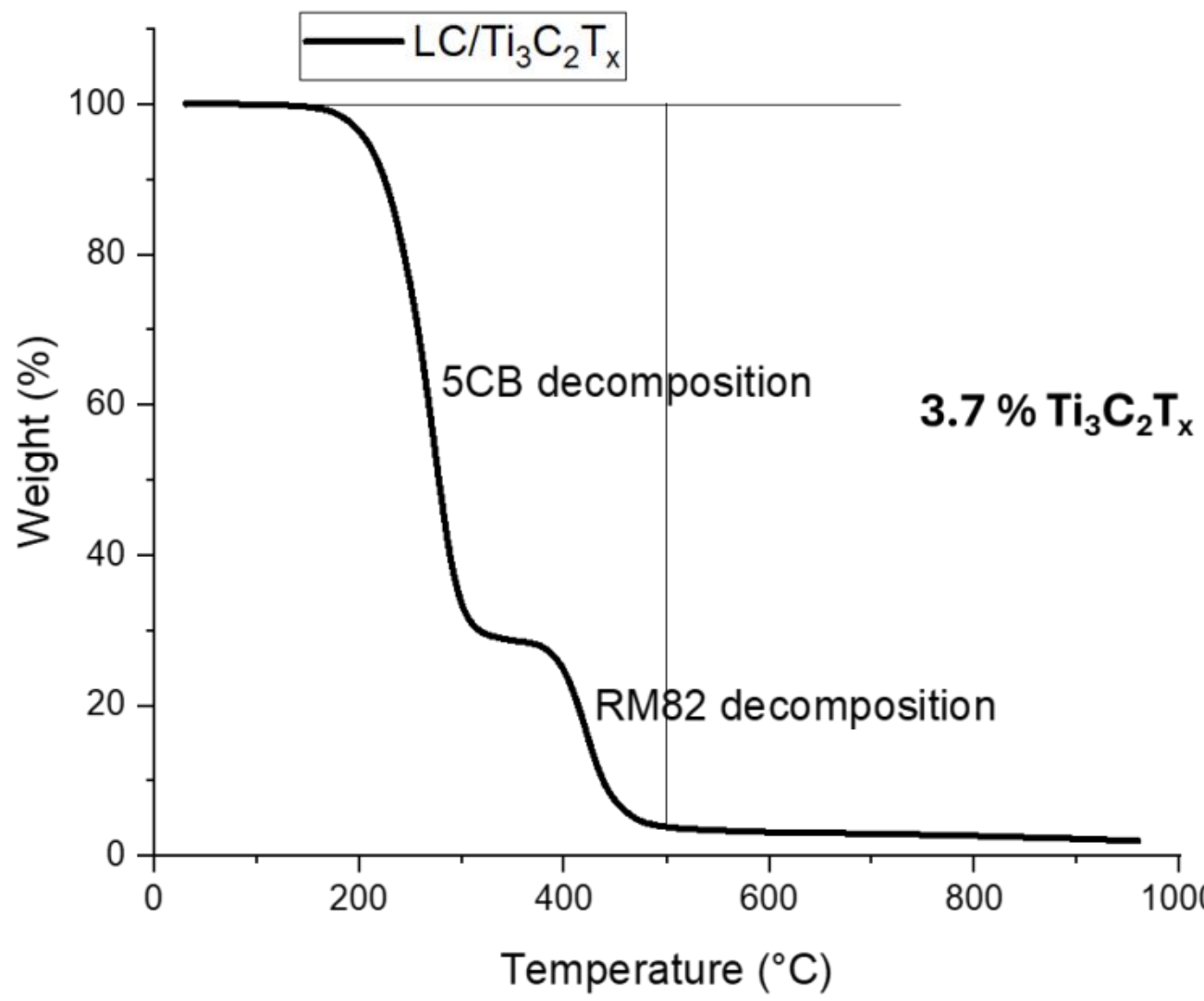

Figure S5. TGA curve of LC/$Ti_3C_2T_x$ composite

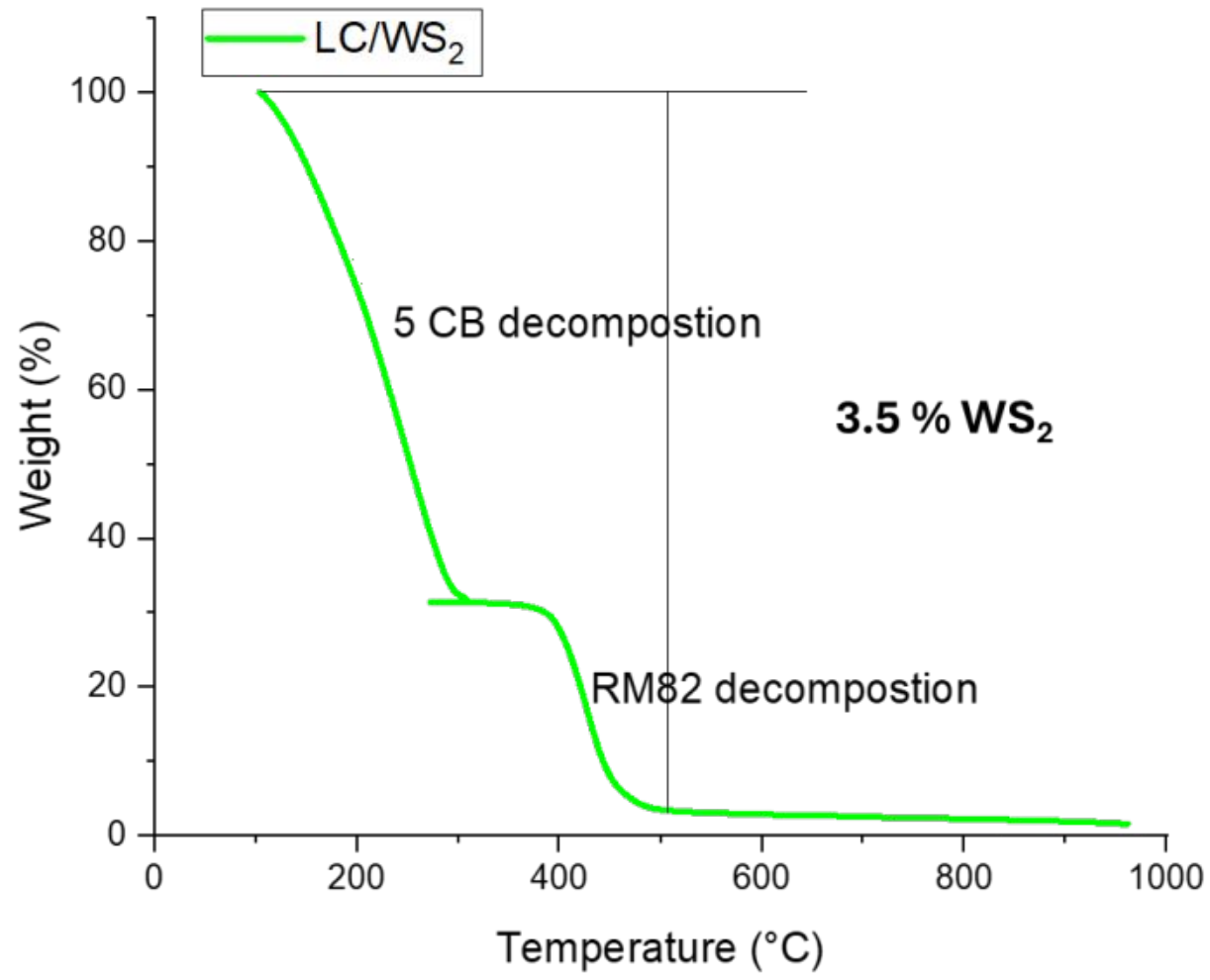

Figure S6. TGA curve of LC/WS_2 composite

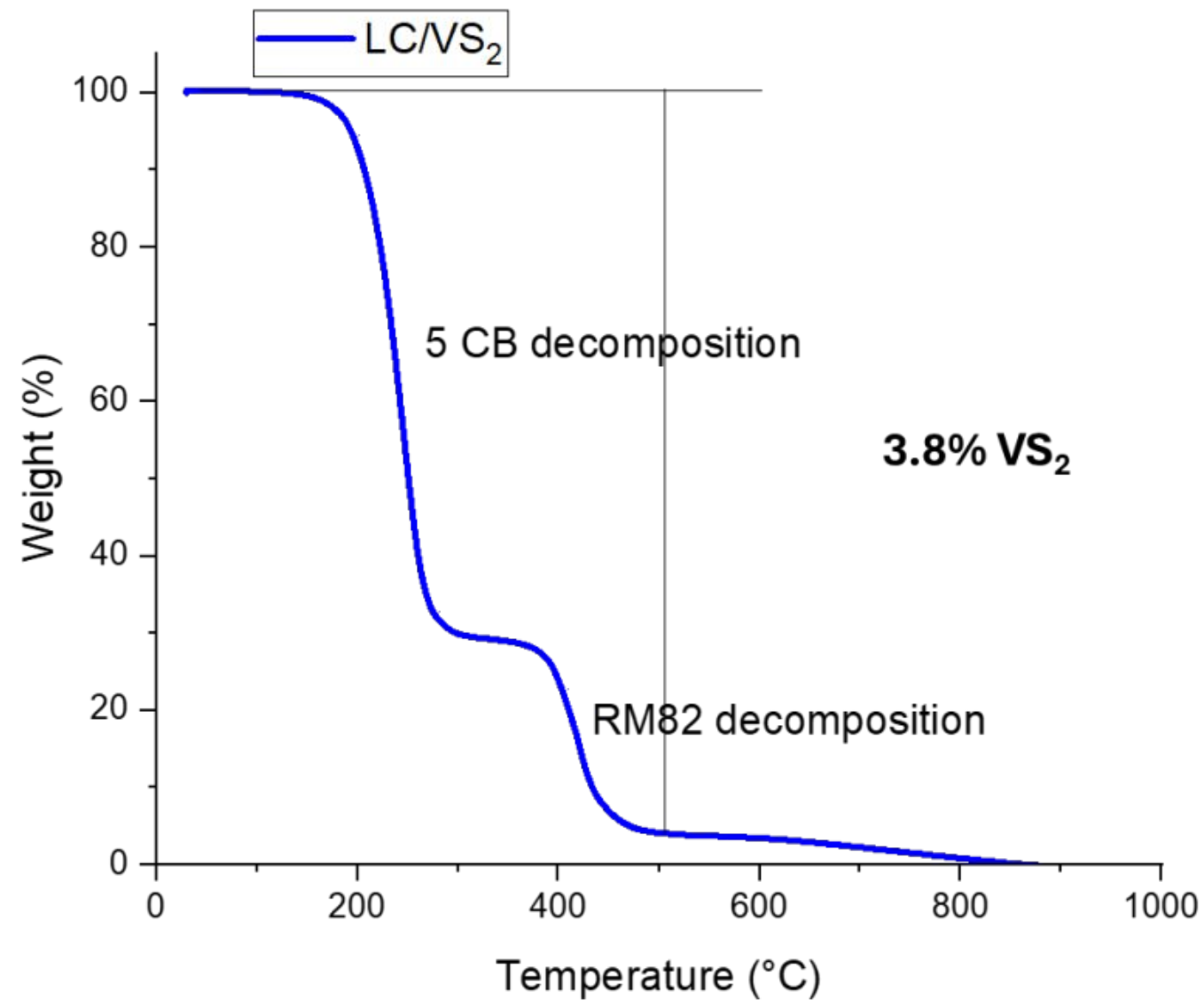

Figure S7. TGA curve of LC/VS_2 composite

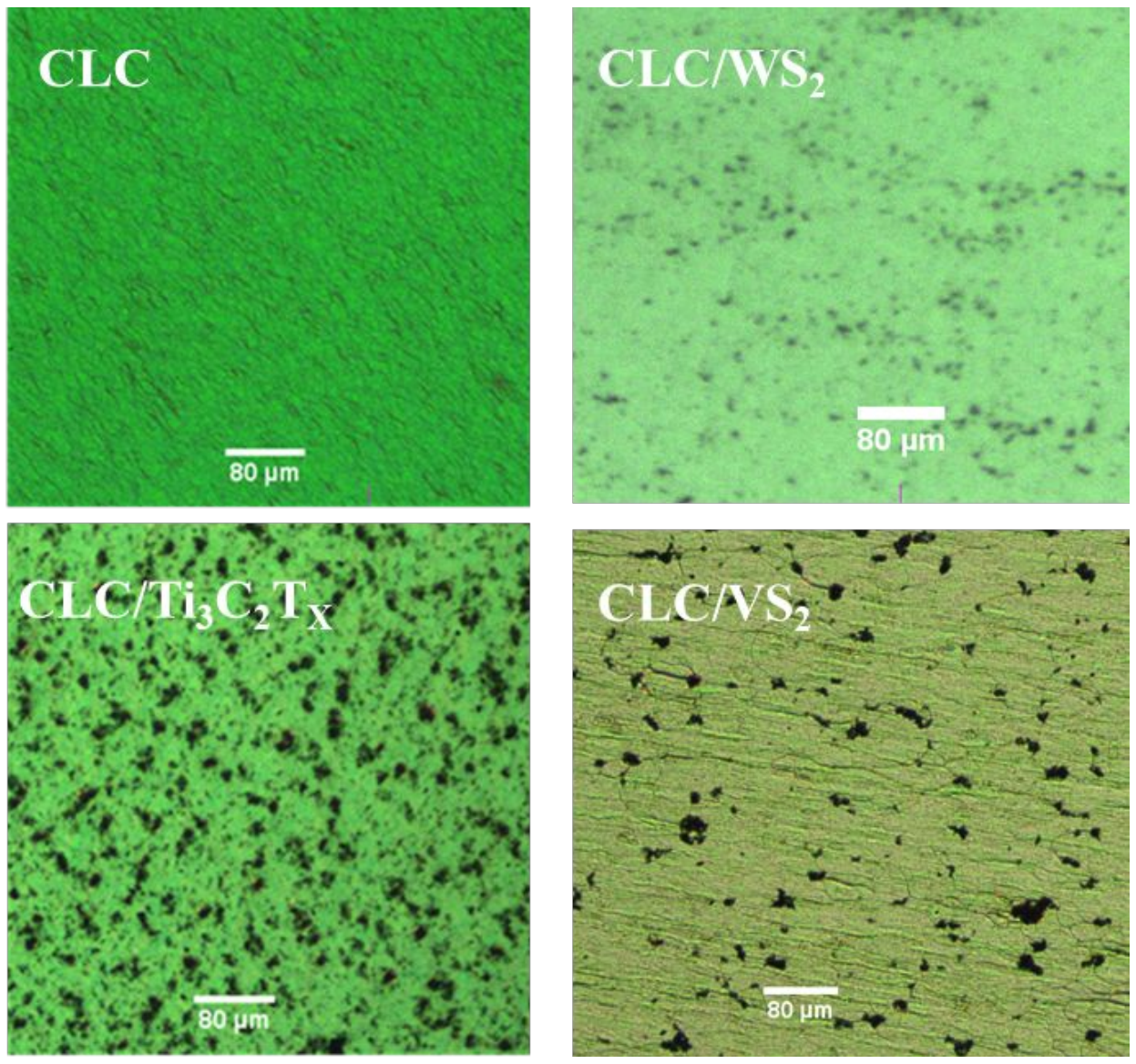

Figure S8. POM images of composites

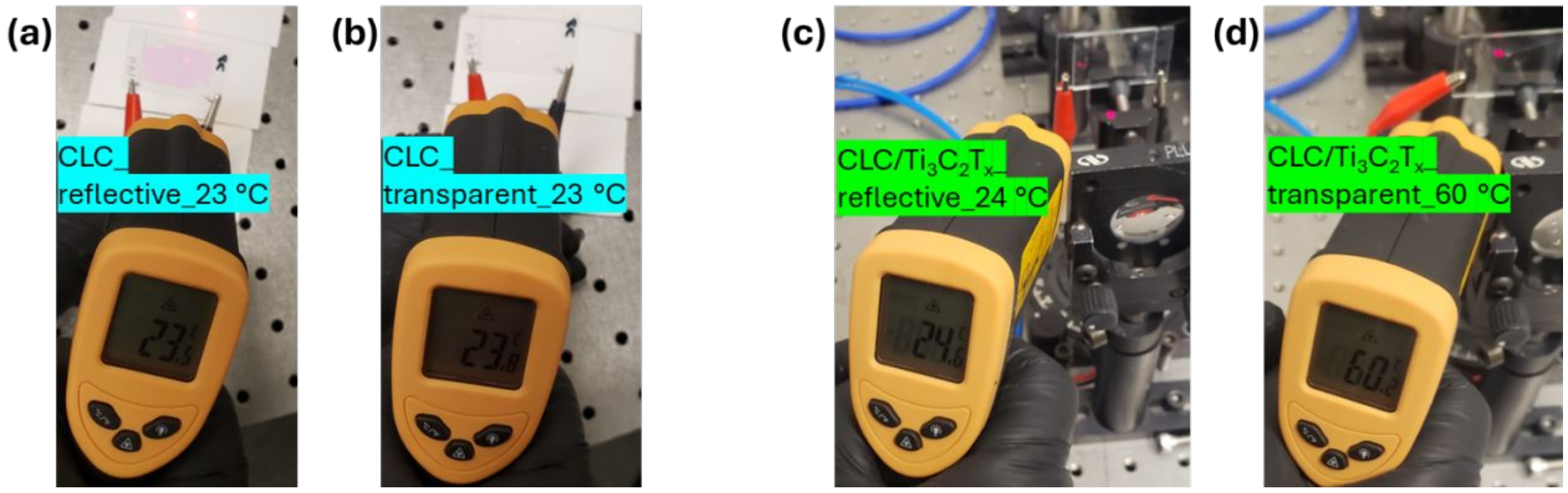

Figure S9. (a, b) Temperature measurements of the CLC cell at the reflective and transparent states respectively. (c, d) Temperature measurement of the CLC/$Ti_3C_2T_x$ cell at the reflective and transparent states respectively.

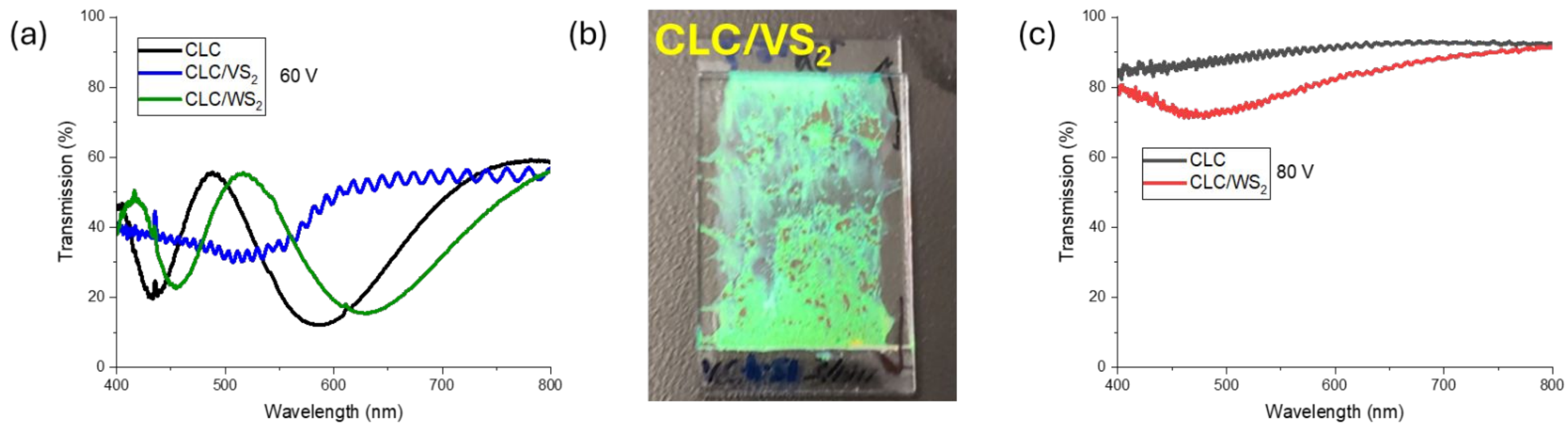

Figure S10. (a) Transmission spectra of CLC, CLC/WS_2 and CLC/VS_2 at 60V. (b) Depiction of CLC/VS_2 cell breakdown. (c) Transmission spectra of CLC and CLC/WS_2 at 80V.

- CLC polymer preparation: S1011,S811 and RM82 (3.4%, 13.8%, 82.8%)
- **Mixture heated on a hot plate at 140C to dissolve components and form CLC phase**
- Methyl methacrylate (0.8 ml) added to disperse CLC mixture and aid in sonication
- 2D material added (e.g. 6 mg $Ti_3C_2T_x$)
- Sonication done for 2.5h, 45-50C
- Mixture kept on hot plate (75C) to prevent crystallization of RM82
- Supernatant taken out and put in another vial (on hot plate)to evaporate excess MMA
- Initiator added (2% with respect to RM82) and mixture sonicated briefly (at 50C)
- Mixture drop casted on substrate on hot plate and temperature increased to 130C to completely evaporate MMA
- Hot plate cooled down to 75C and sample exposed to UV light (365 nm LED) for at least 10 min

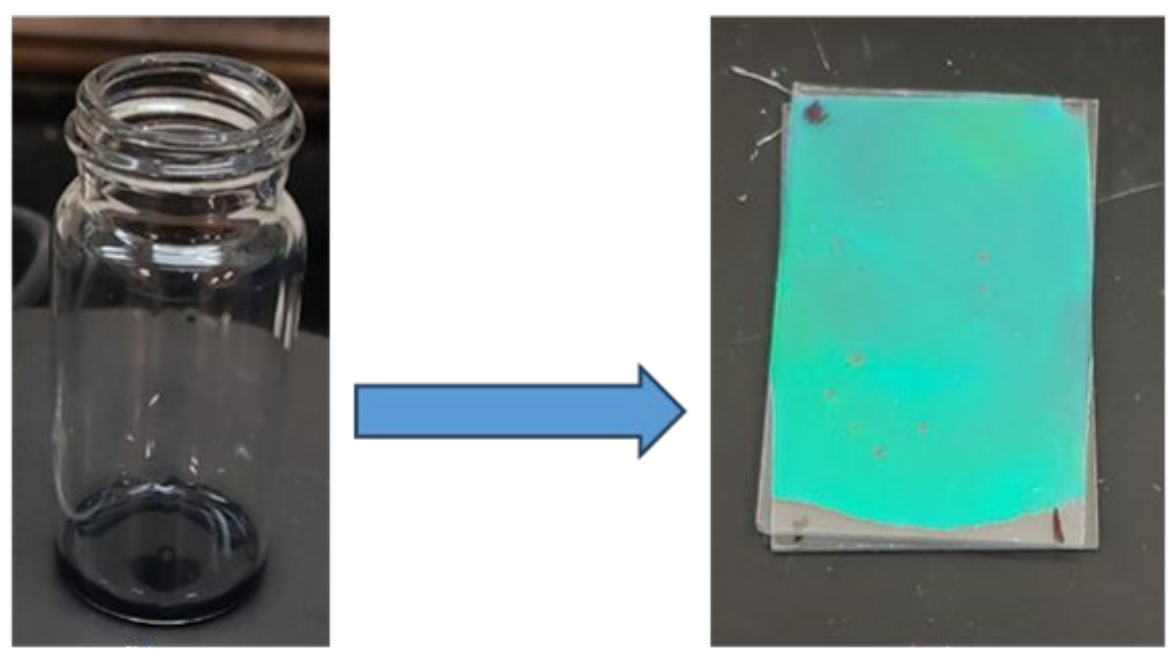

Figure S11. Preparation of CLC Polymer/$Ti_3C_2T_x$ composite
